# A novel methodological approach to simultaneously extract high-quality total RNA and proteins from cortical and trabecular bone

**DOI:** 10.1098/rsob.210387

**Published:** 2022-05-04

**Authors:** Martina Faraldi, Laura Mangiavini, Caterina Conte, Giuseppe Banfi, Nicola Napoli, Giovanni Lombardi

**Affiliations:** ^1^ Laboratory of Experimental Biochemistry and Molecular Biology, IRCCS Istituto Ortopedico Galeazzi, Milan, Italy; ^2^ IRCCS Istituto Ortopedico Galeazzi, Milan, Italy; ^3^ Department of Biomedical Sciences for Health, University of Milan, Milan, Italy; ^4^ Department of Human Sciences and Promotion of the Quality of Life, San Raffaele Roma Open University, Rome, Italy; ^5^ Department of Endocrinology, Nutrition and Metabolic Diseases, IRCCS MultiMedica, Milan, Italy; ^6^ Vita-Salute San Raffaele University, Milan, Italy; ^7^ Unit of Endocrinology and Diabetes, Departmental Faculty of Medicine and Surgery, Campus Bio-Medico University of Rome, Rome, Italy; ^8^ Division of Bone and Mineral Diseases, Washington University in St Louis, St Louis, MO, USA; ^9^ Department of Athletics, Strength and Conditioning, Poznań University of Physical Education, Poznań, Poland

**Keywords:** human cortical and trabecular bone, RNA isolation, protein extraction, RNA and protein integrity and purity, protein and gene expression

## Abstract

Molecular differences between cortical and trabecular bone, of relevance to understanding the pathophysiological basis of bone diseases, can be determined only through effective isolation methods for RNA and proteins. Here we present a TRIzol-based method, which combines bone pulverization and homogenization to extract simultaneously total RNA and proteins from human cortical and trabecular bone from the same carrot. RNA integrity and purity were determined as the 260/280 nm and 260/230 nm absorbance ratios and the 28S/18S rRNA ratio. Protein integrity and quality were evaluated by Coomassie blue staining. Reverse transcription quantitative polymerase chain reaction and immunoblotting for bone-specific genes and proteins were performed to verify the suitability of the isolated material in downstream applications. The 260/280 nm and 260/230 nm absorbance ratios were, on average, less than or equal to 1.8. Bands on agarose gel were consistent with intact RNA, with mean 28S/18S ratios of 1.68 ± 0.35 and 1.88 ± 0.10 for cortical and trabecular bone, respectively. Band patterns after Coomassie blue staining confirmed protein integrity. Successful gene and protein expression analysis, with relevant differences between the two compartments, highlighted the suitability of the material in downstream applications. The method presented here is appropriate and effective for the study of human bone.

## Graphical abstract

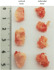

## 1. Introduction

Nucleic acid extraction from bone, and more specifically RNA, represents a big challenge in research [1,2]. Indeed, the study of bone biology has been hindered by several technical issues that have limited the applicability of an expected experimental approach and/or the reliability of the obtained results. The problems that may emerge in the study of *ex vivo* bone samples include: (i) the analysis of full-thickness pieces that consist of both the cortical and the trabecular bony compartments; (ii) low-quality, low-yield, highly fragmented RNA; (iii) separate analysis of RNA and proteins from different samples.

Cortical and trabecular compartments of bone are distinct in terms of macrostructure, protein and mineral distribution/composition and exerted function. The bony function is dependent on the existence of these two distinct compartments. The cortical compartment consists of 70% bone and 30% vascular channels. In contrast, in the trabecular compartment only 20% of the volume is occupied by bone while the remaining space is filled with marrow and fat. The surface-to-volume ratio is much greater in the trabecular than in the cortical bone. During ageing or in the course of disease, cortical porosity increases, thus inducing a gain in the surface-to-volume ratio at the expense of the strength. Trabecular bone senses the mechanical load and transfers it to the cortical bone. Further, trabecular bone contains less calcium and more water than the cortical fraction. The greater exposure of trabecular bone to bone marrow and blood flow and the greater responsiveness to mechanical loading account for the faster turnover rate in the trabecular compartment than in the cortical one [3]. In trabecular bone, resorption takes place along the trabecular surface while in cortical bone it takes place through the formation of tunnels. Bone loss after menopause is faster in trabecular bone, but as cortical bone constitutes 80% of the skeleton the absolute loss is similar for both compartments in the first 10 years; thereafter, the loss of cortical bone prevails [4]. Further, the two compartments have differing sensitivities to hormones and drugs [5]. Despite these relevant aspects, the great majority of studies have focused on either trabecular bone only (especially human studies) or whole bone (especially animal studies) [6].

In contrast to protein extraction, which is not particularly challenging because of its quantitatively relevant presence within the bone matrix, RNA extracted from bone is often of low quality and has undergone some degradation owing to the low number of cells (i.e. low content of nucleic acids) and the high amount of mineralized matrix. A successful extraction of high-quality nucleic acids, and particularly RNA, requires four key steps: (i) to sufficiently reduce (to pulverize) the tissue and to lyse the cells in order to liberate their contents, (ii) to denature the nucleoprotein complexes, (iii) to inactivate the nucleases that may degrade the target molecules, and (iv) to purify the target nucleic acid species [7]. Different strategies have been adopted to limit the degradation of RNA in bone samples; for instance, single-step RNA extraction under near-freezing temperature conditions [8] or homogenization with stabilizing mixtures of guanidinium thiocyanate–phenol–chloroform [9,10]. However, despite these and many other approaches, the yield and quality of the extracted RNA are often below the required standards needed to proceed further with the downstream analyses [2]. Quantification and purity assessment of RNA can be determined spectrophotometrically by reading the optical density of the sample at *λ* = 260 nm, which correlates with the concentration of nucleic acids within the sample; *λ* = 280 nm, which correlates with the concentration of proteins within the sample; and *λ* = 230 nm, which gives a measure of the background and contaminants. Values above 1.8 of the 260/280 nm ratio (i.e. free of protein contamination) and above 2.0 for the 260/230 nm ratio (i.e. free of solvent contamination) indicate the purification of high-quality, low-contamination or pure RNA and the possibility that it can be used in downstream enzyme-based applications [1]. RNA integrity, instead, can be assessed by agarose gel electrophoresis and the visualization of the appropriate resolution of the bands relative to the ribosomal subunits 28S and 18S. An 28S/18S intensity ratio of around 2 is an index of intact RNA [11].

Finally, the possibility of using the same surgical sample to extract, at the same time, RNA and proteins would be desirable as it would allow a direct comparison of transcriptomic and proteomic information [12]. Commercially available reagents allow the implementation of these procedures and, since protein extraction is not a trivial undertaking, the possibility of using the whole biological material, without dividing it for different analyses, increases the RNA yield and, thus, contributes to improving the most difficult phase of the procedure.

To understand the mechanisms of gene and protein regulation in human bone is of pivotal importance in basic, translational and preclinical research since the study of the signalling pathways, the fine regulatory mechanisms and the microenvironment is necessary to unravel molecular alterations that take place in disease (e.g. metabolic bone diseases, osteoporosis, osteoarthritis, osteolysis, osteonecrosis) and to eventually identify possible candidates for targeted therapies. Consequently, a standardized method for extraction and purification of high-quality RNA species and proteins is desirable [2]. Here, we describe a novel approach to the study of human bone biology based on the separation of the cortical and the trabecular compartments from the same bone carrot and the parallel extraction, from the same sample, of high-quality RNA and proteins that can be used for downstream analyses.

## 2. Material and methods

### 2.1. Specimens

**Figure 1.**
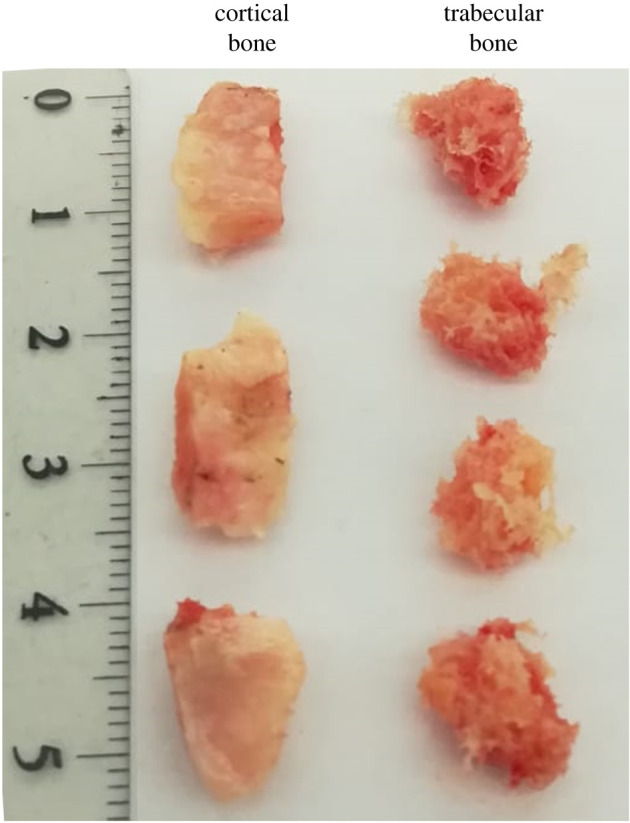
Cortical and trabecular bone pieces. Fragments of cortical and trabecular bone were used in this experimental setting. The scale (ruler on the left) is in centimetres.

Samples were obtained from a total of 10 male and female participants aged 65 years or older, but younger than 85 years, who were recruited from candidates undergoing elective hip replacement surgery at the IRCCS Istituto Ortopedico Galeazzi, Milan, Italy, and enrolled in an observational, cross-sectional study aimed at assessing the differences in several bone parameters between subjects with (body mass index [BMI] ≥30 kg m^−2^) or without (BMI <25 kg m^−2^) obesity. Patients treated with drugs that could affect bone or glucose metabolism, those with a disease known to affect bone or glucose metabolism, alcohol or tobacco users and those with bone metastases or diseases involving the surgical site other than osteoarthritis were excluded. During surgery, bone was harvested from the internal part of the femoral neck with a bone curette. Bone specimens were dissected and cut into smaller pieces with an osteotome after careful removal of the trabecular bone with a Luer bone rongeur. At this level, the division between the two types of bones is particularly neat; thus, isolation of the cortical bone was performed without any difficulties. Particular attention was paid to avoiding contamination between the cortical and trabecular bone (figure 1).

The study protocol complies with the Declaration of Helsinki and was approved by the Ethics Committee of the IRCCS San Raffaele Hospital (protocol VFOWPO, no. 120/2016). All subjects provided informed consent prior to any study procedures.

### 2.2. Tissue homogenization

**Figure 2.**
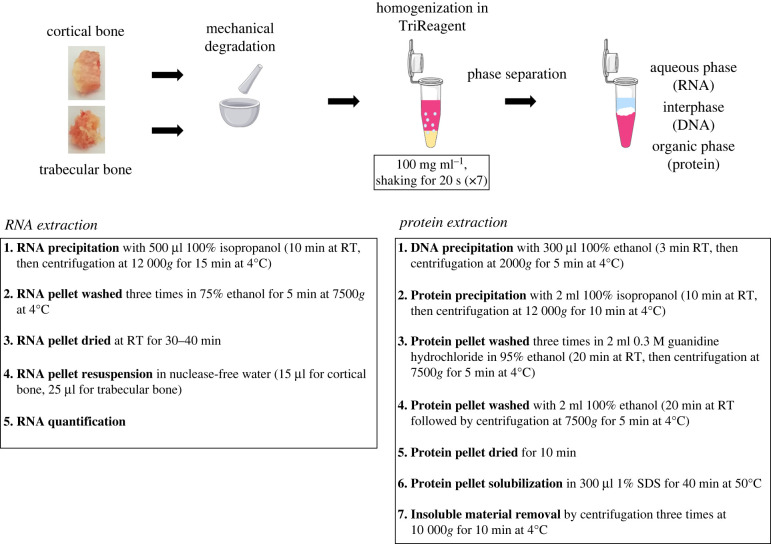
Experimental procedure applied for RNA and protein extraction from bone. Schematic representation of the workflow adopted for RNA and protein extraction from cortical and trabecular bone. Specimens from both cortical and trabecular bone were ground into powder by mechanical degradation in liquid nitrogen and then mechanically homogenized, using ceramic beads, in TRIzol reagent. After separation of the aqueous and the organic phases, containing, respectively, RNA and protein, two different protocols were carried out, as detailed in the figure. RT, room temperature; SDS, sodium dodecyl sulfate.

Once the sample had been collected, any residual soft tissue attached to the bone specimens was removed with scalpel and tweezers. Before freezing at −80°C, bone tissues were washed several times in ice-chilled sterile phosphate-buffered saline, in order to remove blood residues. As shown in figure 2, frozen bones were ground with a pestle and mortar in liquid nitrogen and then stored at −80°C until processing or for 1 month. Four hundred milligrams of bone, reduced to a fine powder, were added to tubes that had been pre-chilled in dry ice and that contained ceramic beads (Precellys Lysing Kit, Tissue Grinding CKmix50_7 mL; Bertin Instrument, Montigny-le-Bretonneux, France). After the addition of 4 ml TriReagent (Ambion Inc., ThermoFisher Scientific, Waltham, MA, USA), according to the recommended proportions (1 ml TriReagent per 100 mg tissue), bone powder was homogenized using a Minilys Homogenizer (Bertin Instrument), at speed 2, for 20 s, seven times. Tubes were kept in ice for 1 min between each homogenization cycle in order to allow the sample, beads and TriReagent to chill.

### 2.3. Cell cultures

SaOS-2 cells were cultured in Dulbecco's modified Eagle's medium with 10% fetal bovine serum, 2 mM glutamine, 1 × 10^5^ U L^−1^ penicillin and 0.1 mg l^−1^ streptomycin in a humidified incubator at 37°C and 5% CO_2_. Cells were harvested with TriReagent after 5 days of culture and stored at −80°C until RNA and protein extraction.

### 2.4. RNA extraction, quantification and quality assessment

RNA extraction was performed immediately after bone homogenization. As detailed in figure 2, after homogenization, tubes were kept in ice, in a vertical position for 5 min, to allow the residual bone powder to sediment at the bottom of the tube. The TriReagent supernatant was then transferred to a 1.5 ml nuclease-free tube (1 ml of TriReagent in a 1.5 ml tube), and RNA extraction was performed as recommended by the TriReagent protocol (Ambion Inc., ThermoFisher Scientific, Waltham, MA, USA) (figure 2). Two hundred microlitres of chloroform (Sigma, St Louis, MO, USA) (200 µl chloroform per 1 ml TriReagent (1 : 5)) was added to each sample. Tubes were manually shaken and, after 3 min at room temperature and after being centrifuged at 12 000*g* for 15 min at 4°C to allow phase separation, the aqueous phase was used for RNA extraction, while the phenol–ethanol phase was used for protein extraction (see Protein extraction, quantification and quality assessment). The aqueous phase from each tube was transferred to a new tube, in which 500 µl of 100% isopropanol was added (500 µl per 1 ml TriReagent (1 : 2)). After inverting four times and resting at room temperature for 10 min, tubes were centrifuged at 12 000*g* for 15 min at 4°C; the supernatant was discarded whereas the pellet was washed three times in 75% ethanol for 5 min at 7500*g* at 4°C. After being dried, the pellet was resuspended in nuclease-free water (15 µl for pellets derived from cortical bone and 25 µl for pellets derived from trabecular bone). The RNA concentration was quantified on a NanoDrop spectrophotometer (ThermoFisher Scientific). RNA purity was assayed by assessing the 260/280 nm and 260/230 nm absorbance ratios (with optimal values >1.8) [13]. RNA integrity was evaluated electrophoretically on 1% agarose gel and by subsequently calculating the ratio of the intensity of the bands relative to 28S and 18S rRNA [14]. The analysis of the 28S and 18S peaks was performed using Image Lab 6.0 software (Bio-Rad Laboratories, Hercules, CA, USA). RNA isolation from SaOS-2 cells was performed as recommended by the TriReagent protocol (Ambion Inc.), and as previously described, avoiding the homogenization step. RNA from SaOS-2 cells was used as an internal control. After these quality control checks, RNA samples were frozen at −80°C until gene expression analysis.

### 2.5. Gene expression

Total RNA was submitted to digestion with DNase I (Invitrogen, ThermoFisher Scientific) to eliminate any potential DNA contamination, and then reverse transcribed using the iScript cDNA Synthesis Kit (Bio-Rad Laboratories). Reverse transciption quantitative polymerase chain reaction (RT-qPCR) was performed on a QuantStudio 12 K Flex Real-Time PCR System (Applied Biosystem, ThermoFisher Scientific), using the TaqMan Gene Expression Master Mix and pre-made 6-carboxyfluorescein (FAM)-labelled TaqMan assay for sclerostin (*SOST*, Hs00228830_m1), periostin (*POSTN*, Hs01566750_m1) and osteopontin (*SPP1*, Hs00959010_m1) (ThermoFisher Scientific). As potential reference genes, β-actin (*ACTB*, Hs99999903_m1), glyceraldehyde 3-phosphate dehydrogenase (*GAPDH*, Hs99999905_m1) and rRNA 18S (*18S*, Hs99999901_s1) were tested. RT-qPCR was performed based on the following protocol: polymerase activation for 2 min at 50°C, followed by a second step at 95°C for 10 min, 40 amplification cycles at 95°C for 15 s and 60°C for 60 s. Results were reported as quantification cycle (*C*_q_) values. The expression stability of the potential reference genes was assessed by considering the relative expression of these genes as the difference between the *C*_q_ of a reference gene in a sample and the geometrical mean of the *C*_q_ of ACTB, GAPDH and 18S in all samples. The expression stability of these genes was analysed using the NormFinder algorithms and GAPDH was found to be the most stable one. The relative expression of SPP1, POSTN and SOST was than calculated by the 2^−ΔΔ*C*q^ method, using GAPDH as a reference gene.

### 2.6. Protein extraction, quantification and quality assessment

Proteins were extracted from bone specimens (cortical and trabecular) and SaOS-2 cells following the TriReagent protocol (Ambion Inc.) (figure 2). After phases separation, in order to remove any aqueous phase overlying the interphase and DNA contamination, 300 µl of 100% ethanol (300 µl ethanol per 1 ml TriReagent (1 : 3.3)) was added to each tube, followed by multiple tube inversions. After 3 min rest at room temperature, samples were centrifuged for 5 min at 2000*g* at 4°C and the protein fraction-containing supernatants were collected in new tubes and stored at −80°C, according to the manufacturer's instructions. Once thawed, the phenol–ethanol phases were incubated with 1.5 ml of 100% isopropanol (1.5 ml isopropanol per 1 ml TriReagent (1.5 : 1)), after inverting a few times, for 10 min at room temperature; then the suspensions were centrifuged for 10 min at 12 000*g* at 4°C. The obtained pellets were washed three times with 2 ml of 0.3 M guanidine hydrochloride in 95% ethanol (2 ml guanidine hydrochloride 0.3 M in 95% ethanol per 1 ml TriReagent (2 : 1)), left to incubate for 20 min at room temperature and then centrifuged for 5 min at 7500*g* at 4°C. Pellets were washed in 2 ml of 100% ethanol, left to incubate for 20 min at room temperature and then centrifuged for 5 min at 7500*g* at 4°C. Protein pellets were left to rest for 10 min at room temperature to dry; thereafter, pellets were resuspended in 300 µl (pellets from bone specimens) and 70 µl (pellets from SaOS-2 cells) of 1% sodium dodecyl sulfate (SDS) and incubated for 40 min at 50°C. Suspensions were centrifuged three times for 10 min each at 10 000*g* at 4°C in order to remove insoluble materials. Between each centrifugation, the supernatants were transferred to new tubes. The supernatant obtained from the final centrifugation was stored at −80°C. Proteins were quantified using a Pierce BCA Protein Assay Kit (ThermoFisher Scientific) and the absorbance was measured with a Victor multilabel plate reader (Perkin Elmer, Waltham, MA, USA) at *λ* = 540 nm. The protein concentration was determined from interpolation of the absorbance reading on a linear regression standard curve. Bio-Safe Coomassie Staining (Bio-Rad Laboratories) was used to assess the protein integrity with 12% sodium dodecyl sulfate–polyacrylamide gel electrophoresis (SDS-PAGE). For staining, gels were incubated for 1 h with Bio-Safe Coomassie Staining and then washed with distilled water overnight at room temperature.

### 2.7. Western blotting

Ten micrograms of extracted proteins were separated on 12% SDS-PAGE. Proteins were then transferred onto a polyvinylidene fluoride (PVDF) membrane (Bio-Rad Laboratories) and activated in methanol for 30 s. After blocking with 5% not-fat dried milk or 5% bovine serum albumin in 0.1% Tris-buffered saline with 0.1% Tween^®^ 20 detergent, for 1 h at room temperature, membranes were incubated overnight at 4°C with with anti-sclerostin (SOST) (AP13236PU-N) (Origene, Rockville, MD, USA), anti-osteopontin (OPN) (ab8448; RRID: AB_306566) (Abcam, Cambridge, UK), anti-osteonectin (SPARC) (cs5420; RRID: AB_10692794) (Cell Signaling, Danvers, MA, USA), anti-osteocalcin (OCN) (ab133612) (Abcam, Cambridge, UK), anti-vinculin (ab129002; RRID: AB_11144129) (Abcam), anti-β-actin (TA811000) (Origene) or anti-glyceraldehyde 3-phosphate dehydrogenase (GAPDH) (ab9485; RRID: AB_307275) (Abcam) antibody. After incubation with secondary antibodies, goat anti-mouse immunglobulin G (IgG) (H + L)-horseradish peroxidase (HRP) conjugate (170–6516; RRID: AB_11125547) and goat anti-rabbit IgG (H + L)-HRP conjugate (170–6515; RRID: AB_11125142) (Bio-Rad Laboratories) for 1 h at room temperature, protein detection was performed using Clarity-Max Enhanced Chemioluminescence (ECL) (Bio-Rad Laboratories). Signals were detected with ChemiDoc (Bio-Rad Laboratories). To test the best loading control for protein analysis, i.e. the one with the lowest variability, the coefficients of variation (CoV) of the densitometry values for β-actin, GAPDH and vinculin were compared.

### 2.8. Statistical analysis

Statistical analysis was performed with Prism^®^ v. 6.01 (GraphPad Software Inc., La Jolla, CA, USA). The normal distribution of data was tested through the D'Agostino Pearson normality test. Comparison of gene and protein expression between cortical and trabecular bone was performed through the Mann–Witney test. Differences were considered to be statistically significant if *p*-values were <0.05.

## 3. Results

### 3.1. RNA quantity, quality and integrity

**Figure 3.**
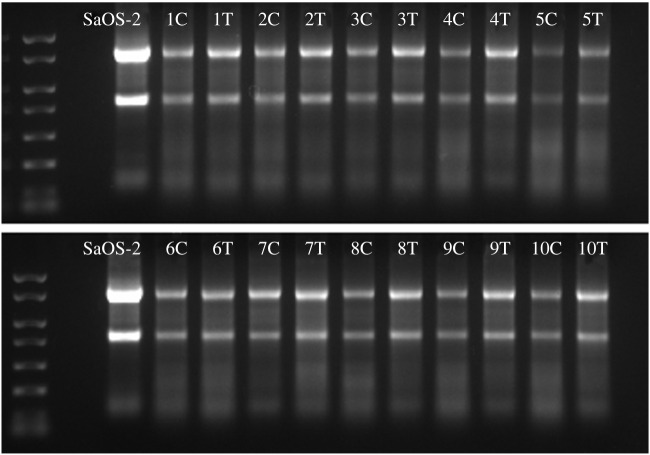
RNA from SaOS-2 cells and cortical and trabecular bone. Agarose gel (1%) of RNA isolated from SaOS-2 cells and cortical (C) and trabecular (T) bone.

Total RNA was isolated from cortical and trabecular bone (figure 1). As a control for RNA isolation using a standard protocol without homogenization, total RNA was extracted from cells (SaOS-2). Total RNA concentrations are shown in table 1. The yield from trabecular bone (1639.08 ± 414.86 ng µl^−1^) was higher than that from cortical bone (731.86 ± 418.91 ng µl^−1^). RNA purity, i.e. samples free from protein and organic compound contaminations, was determined spectrophotometrically by means of the 260/280 nm and 260/230 nm absorbance ratios. As shown in table 1, ratios greater than or equal to 1.8, in most samples, similar to those obtained for RNA extracted from SaOS-2 cells, indicate a high-quality level of RNA; in only five samples the 260/230 nm ratio was lower, but very close to 1.8. To assess RNA integrity, intact 28S and 18S rRNA was assayed on agarose gel electrophoresis (figure 3 and electronic supplementary material, figure S1) by calculating the 28S/18S band intensity ratio (table 1). The readings revealed no degradation signs and intact RNA in all samples. The mean ± s.d. of the 28S/18S intensity ratios was 1.68 ± 0.35, 1.88 ± 0.10 and 2.00 ± 0.02 for cortical bone, trabecular bone and SaOS-2 cells, respectively.

**Table 1.** Concentration, quality and integrity of cortical and trabecular bone RNA.

sample	ng µl^−1^	ng/100 mg	260/280	260/230	28S/18S
SaOS-2 a	1318.05	/	1.98	1.91	1.98
SaOS-2 b	931.33	/	1.96	1.98	2.02
1c	727.07	10 906.05	1.95	2.01	1.62
1t	1257.39	31 434.75	1.96	2.09	1.96
2c	477.18	7157.70	1.93	1.82	1.69
2t	1244.57	31 114.25	1.95	2.20	1.89
3c	595.48	8932.20	1.96	1.93	1.61
3t	1251.89	31 297.25	1.93	2.07	1.85
4c	675.23	10 128.45	2.02	1.99	1.39
4t	1734.36	43 359.00	1.97	2.02	1.88
5c	567.18	8507.70	1.98	1.80	1.50
5t	1766.33	44 158.25	1.98	1.97	1.93
6c	609.76	9146.40	1.92	1.67	1.60
6t	1891.87	47 296.75	1.98	1.76	1.64
7c	1884.21	28 263.15	1.95	1.78	2.63
7t	2344.07	58 601.75	1.96	1.70	1.82
8c	384.62	5769.30	1.87	1.81	1.54
8t	1143.34	28 583.50	1.97	1.81	1.91
9c	702.04	10 530.60	2.03	2.06	1.53
9t	1603.53	40 088.25	1.97	2.30	1.98
10c	695.79	10 436.85	2.03	1.77	1.56
10t	2153.44	53 836.00	1.97	2.12	1.94

Table 1 shows the value of the RNA concentration (ng µl^−1^), RNA (ng) per unit of tissue weight (100 mg), 260/280 nm and 260/230 nm absorbance ratios measured using a NanoDrop spectrophotometer (ThermoFisher Scientific, Waltham, MA, USA) and the 28S/18S ribosomal RNA ratio using Image Lab 6.0 software (Bio-Rad Laboratories, Hercules, CA, USA). As an index of RNA purity, the 260/280 nm and 260/230 nm absorbance ratios were used; as an index of RNA integrity, the 28S/18S ribosomal RNA ratio was used.

### 3.2. Gene expression analysis in cortical and trabecular bone

To test the applicability of the isolated RNA in downstream molecular biology analyses, the expression of bone-specific genes was analysed through RT-qPCR. *C*_q_ values of genes encoding for *SPP1*, *SOST*, *POSTN*, *ACTB*, *GAPDH* and *18S* are shown in table 2.

**Table 2.** Quantification cycle (*C*_q_) values of *ACTB*, *GAPDH*, *18S*, *SOST*, *POSTN* and *SPP1* of SaOS-2 cells and cortical and trabecular bone from gene expression analyses through RT-qPCR. c, cortical bone; t, trabecular bone; *ACTB*, β*-*actin; *GAPDH,* glyceraldehyde 3-phosphate dehydrogenase; *18S*, 18S ribosomal RNA*; SOST,* sclerostin; *POSTN*, periostin; *SPP1*, osteopontin.

sample	*ACTB*	*GAPDH*	*18S*	*SOST*	*POSTN*	*SPP1*
SaOS-2 a	31.70	29.29	24.10	37.43	37.81	34.32
SaOS-2 b	30.15	28.01	24.52	36.41	35.80	32.85
1c	>38	34.76	32.73	>38	35.96	27.66
1t	35.32	32.23	30.26	>38	34.62	27.97
2c	>38	35.44	32.57	>38	36.53	27.63
2t	36.57	32.39	30.68	>38	35.05	28.25
3c	>38	35.04	32.42	>38	34.75	27.26
3t	35.16	31.70	27.25	>38	33.94	28.42
4c	37.56	33.03	28.65	36.14	32.76	26.83
4t	29.42	28.48	23.72	>38	32.33	26.86
5c	37.72	33.67	25.12	35.71	30.27	24.09
5t	36.65	33.04	27.90	>38	34.54	28.02
6c	34.68	32.12	27.81	36.50	32.15	23.91
6t	33.71	32.09	27.79	>38	33.80	25.97
7c	34.32	31.48	27.94	>38	31.43	24.15
7t	35.40	33.67	30.20	>38	28.59	27.95
8c	34.54	32.48	28.75	36.40	30.68	23.10
8t	33.43	30.51	27.17	>38	34.37	27.68
9c	>38	33.38	29.64	36.85	32.51	27.13
9t	31.16	29.39	24.16	>38	33.43	27.17
10c	37.03	32.80	28.35	36.32	32.64	26.46
10t	30.76	29.52	24.00	>38	33.21	27.34

**Figure 4.**
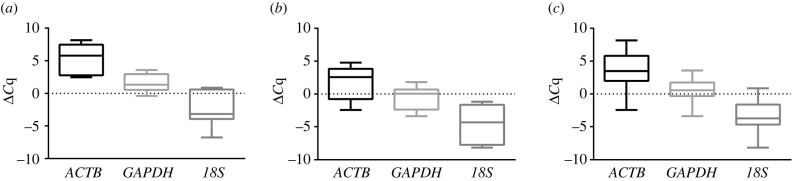
Expression profile of *ACTB, GAPDH* and *18S* in cortical and trabecular bone. Relative expression of *ACTB*, *GAPDH* and *18S* in cortical bone samples (*a*), trabecular bone samples (*b*) and both cortical and trabecular samples (*c*). The relative expression for each gene is calculated as the difference between the *C*_q_ of a reference gene in a sample and the geometrical mean of the *C*_q_ of *ACTB*, *GAPDH* and *18S* of all samples. Data are shown as the 5th–95th percentiles.

*ACTB*, *GAPDH* and *18S* were tested as reference genes. The expression level of these genes, in term of *ΔC*_q_, revealed that the distribution width of the *GAPDH* level was more strict than those of *ACTB* and *18S* considering cortical and trabecular bone samples, both separately and matched (figure 4).

**Figure 5.**
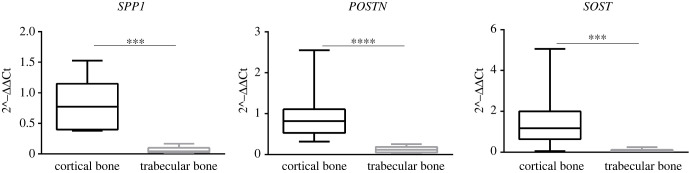
*SPP1*, *SOST* and *POSTN* expression levels in cortical and trabecular bone. Expression profiles of *SPP1*, *SOST* and *POSTN* in cortical and trabecular bone. Statistical analyses of the expression profiles in cortical and trabecular bone were performed through the Mann–Whitney test. *p*-value < 0.05 indicates a statistically significant comparison. All data are shown as the 5th–95th percentiles. Statistical analysis was performed with Prism v. 6.01 (GraphPad Software).

Moreover, according to the NormFinder analysis, *GAPDH* was the most reliable reference gene in this specific experimental setting (*GAPDH* s.d. = 0.67; *ACTB* and *18S* s.d. > 1). Gene expression analysis highlighted relevant differences between cortical and trabecular bone with *SPP1*, *SOST* and *POSTN* being more expressed in the cortical than in the trabecular fraction (figure 5).

### 3.3. Protein quantity, quality and integrity

Proteins were extracted from cortical and trabecular bone and from SaOS-2 cells as a control.

The mean values of the cortical and trabecular bone protein concentrations were 1301.12 ± 589.93 ng µl^−1^ and 1067.15 ± 339.82 ng µl^−1^, respectively (table 3).

**Table 3.** Concentration of SaOS-2 cells and cortical and trabecular bone proteins.

sample		μg ml^−1^		μg/100 mg	
		cortical bone	trabecular bone	cortical bone	trabecular bone
SaOS-2	1051.30				
1		791.89	1240.04	237 567	372 012
2		1306.89	759.80	392 067	227 940
3		1154.19	767.91	346 257	230 373
4		1489.35	1805.23	446 805	541 569
5		2200.75	1173.39	660 225	352 017
6		443.74	1137.97	133 122	341 391
7		1398.65	1332.69	419 595	399 807
8		376.63	885.77	112 989	265 731
9		2306.05	762.63	691 815	228 789
10		895.00	806.07	268 500	241 821

Table 3 shows the protein concentration values in micrograms per millilitre and micrograms of protein per unit of tissue weight (100 mg) of both cortical and trabecular bone. The concentration (μg ml^−1^) of proteins extracted from SaOS-2 cells is also reported.

**Figure 6.**
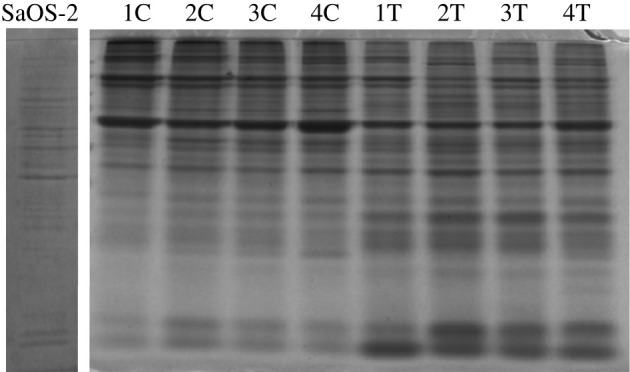
Protein profile of SaOS-2 cells and cortical and trabecular bone. Coomassie blue staining of protein extracted from SaOS-2 cells and cortical (C) and trabecular (T) bone. For each sample, 10 ng of protein was separated on 12% acrylamide gel.

Staining with Coomassie blue has not revealed any degradation in proteins obtained after bone homogenization, as in SaOS-2 cells (figure 6 and electronic supplementary material, figure S2). Importantly, the staining has highlighted a different protein pattern between cortical and trabecular bone (figure 6 and electronic supplementary material, figure S2).

### 3.4. Expression of cortical and trabecular bone proteins

**Figure 7.**
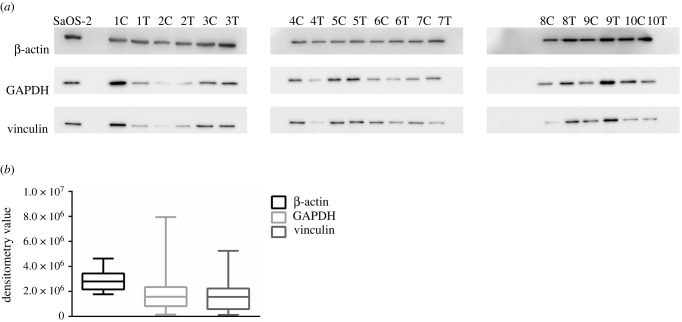
Expression profile of loading control protein β-actin, GAPDH and vinculin in SaOS-2 cells and cortical and trabecular bone. (*a*) Immunoblot analysis of expression levels of loading control protein β-actin, GAPDH and vinculin in SaOS-2 cells and cortical and trabecular bone. (*b*) Densitometry analysis of β-actin, GAPDH and vinculin in SaOS-2 cells and cortical (C) and trabecular (T) bone. Data are shown as the 5th–95th percentiles.

β-actin, GAPDH and vinculin were tested as potential loading control for cortical and trabecular bone proteins. As shown in figure 7 and electronic supplementary material, figure S3, β-actin had the lowest variability among all the analysed samples: the distribution of densitometry values of GAPDH and vinculin was much more scattered than the distribution of β-actin, considering all samples together.

This result was confirmed by the CoV of densitometry values, as shown in table 4. The CoV of actin (0.27) were significantly lower than those of GAPDH (1.02) and vinculin (0.98).

**Table 4.** Protein expression stability of β-actin, GAPDH and vinculin.

CoV			
	cortical bone	trabecular bone	cortical and trabecular bone
β-actin	0.21	0.29	0.27
GAPDH	1.03	1.07	1.02
vinculin	0.75	0.98	0.98

Expression stability of β-actin, GAPDH and vinculin in cortical bone samples, trabecular bone samples and cortical and trabecular bone samples. Expression stability was defined as the coefficient of variation (CoV) of the densitometry values for β-actin, GAPDH and vinculin.

**Figure 8.**
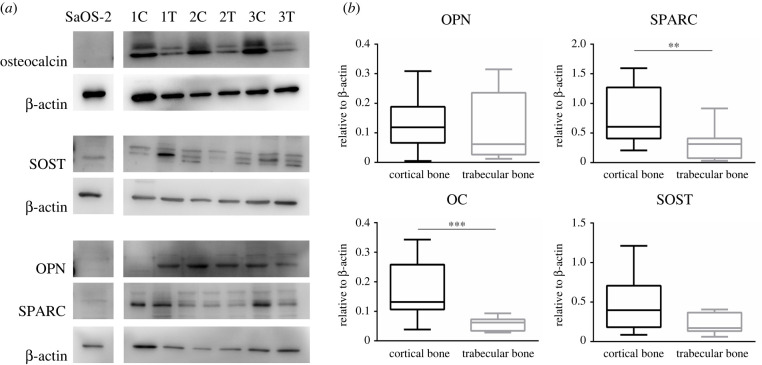
Expression profiles of OPN, sclerostin, OC and SPARC in SaOS-2 cells and cortical and trabecular bone. (*a*) Immunoblot analysis of expression levels of OPN, sclerostin, OC and SPARC in SaOS-2 cells and cortical (C) and trabecular (T) bone. (*b*) Densitometry analysis of OPN, sclerostin, OC and SPARC in SaOS-2 cells and cortical and trabecular bone. Data are shown as the 5th–95th percentiles. Statistical analysis of expression profiles in cortical and trabecular bone were performed through the Mann–Whitney test. *p*-value < 0.05 indicates statistically significant comparison. All data are shown as the 5th–95th percentiles. Statistical analysis was performed with Prism v. 6.01 (GraphPad Software).

Expression of bone-specific proteins such as OPN, SPARC, OC and SOST was assayed in both cortical and trabecular bone (figure 8 and electronic supplementary material, figures S4–S6). While no differences were observed for SOST and OPN, the expression of OC and SPARC was higher in cortical bone than in trabecular bone.

## 4. Discussion

Improving the methods for total RNA and protein extraction from human bone could enhance the potential of studies aimed at understanding relevant aspects of bone biology, and contribute to the discovery and validation of reliable biomarkers of bone disease. Being a mineralized tissue, which is very difficult to handle, molecular analysis of human bone is more challenging than the corresponding analysis in soft tissues [15]. Indeed, current studies on RNA and protein extraction from bone are mainly focused on experimental animal models (e.g. mice, rats), which are easier to handle, rather than on human bone [2,8,16–18]. Studies using TRIzol-based protocols to extract proteins are more common in the case of soft tissues or cell lines [12,19–21]; moreover, protein extraction methods were mainly propaedeutic to proteomic analysis that is based, principally, on bone decalcification followed by protein solubilization and, hence, do not rely on the analysis of native proteins [22–25].

In this study, we describe a TRIzol-based total RNA and protein extraction method from both human cortical and trabecular bone. This is a multistep approach that starts with bone pulverization and homogenization with beads followed by total RNA and protein extraction, from the same sample. The requirement of the pulverization step is fundamental in RNA isolation from tissue as hard as bone to isolate high-quality RNA, as has been demonstrated in a study by Wilson *et al*. [15]. The coupled extraction of total RNA and protein represents another crucial step in molecular analysis and optimizes the yield in cases of small samples or in cases of limited availability of surgical samples. Moreover, the possibility to extract RNA and proteins from the same sample enables a direct and parallel comparison between transcriptome and proteome for a better understanding of the molecular mechanisms underlying the pathogenesis of bone diseases. Although studies have detailed the extraction of RNA or proteins from bone using TRIzol reagent, our current report represents the first one describing a strategy for the simultaneous extraction of both molecules from human bone. This study also evaluated the application of this strategy to the two fractions, cortical and trabecular, obtained from the same carrot, highlighting the potential of assessing molecular differences in these two compartments. These measures may improve the results detailed in previous studies, as in Piccoli *et al.* [26]. This study detailed whether the alteration of sclerostin gene expression and of accumulation of advanced glycation end-product (AGE) can affect bone turnover and fracture risk in patients with type 2 diabetes (T2D). The analysis on bone formation and Wnt pathway markers, together with the analyses on bone microarchitecture and strength, were performed on trabecular bone of postmenopausal women with or without T2D who were undergoing hip replacement surgery. Results showed a decrease in gene expression of the bone-forming transcription factor RUNX2 and an increase in sclerostin gene expression in patients with T2D. The assessment of these bone markers also in cortical bone, besides highlighting differences in the two bone compartments, would provide new insights into bone fracture risk in patients with T2D [26].

The main challenge in performing molecular analysis on RNA and protein is represented by the preservation of their integrity and, indeed, our first goal was to avoid any degradation. Because of this, each step was performed at temperatures near freezing: bone pulverization was conducted in liquid nitrogen and bone homogenization was performed using pre-chilled materials and leaving samples for 1 min in ice between each homogenization cycle. To assess the RNA integrity, the 28S and 18S rRNA bands on agarose gel were analysed and the 28S/18S intensity ratio was calculated for all samples. The ratio values obtained for cortical (1.68 ± 0.35) and trabecular (1.88 ± 0.10) bone tended to the optimal value, i.e. 2 [11]. It is noteworthy that the 28S/18S ratio for cortical bone tended to be lower than that for trabecular bone, probably because cortical bone is harder to pulverize and homogenize than trabecular bone. As shown by our results, the measures adopted in our multistep extraction protocol allowed us to obtain high-quality RNA. This is in contrast with the results from Carter *et al*. [8], who pointed out the importance of performing mouse bone pulverization and RNA extraction in a single step [8]. RNA integrity can also be ensured by storing samples in an RNA stabilization reagent such as RNAlater. A recent study by Pedersen *et al*. [17] has demonstrated that incubation of mouse bone (femur shaft, femur bone marrow and vertebral bone) with RNAlater improves RNA quality [17]. However, although we did not have a comparison with an RNAlater-stored counterpart, our results revealed that extracting intact RNA without the use of any RNA stabilization reagents is possible and reliable.

To assess protein integrity, Coomassie blue staining of total protein extracts following SDS-PAGE was performed and, as for RNA, the distribution patterns of bands revealed protein integrity in both the cortical and the trabecular compartments. These results suggest that bead-based bone homogenization can be effectively applied to extract RNA and proteins that are qualitatively comparable to those obtained from SaOS-2 cells, where no homogenization step is required.

Besides RNA integrity, an assessment of RNA purity is also needed to perform gene expression analysis and a highly pure material is required to obtain meaningful and reliable results. Indeed, contamination due to phenolic compounds, used during the extraction process, or proteins could interfere with the downstream RT-qPCR-based applications, compromising experimental results [13]. The spectrophotometric assessment of RNA purity by the ratio of the optical readings at 260/280 nm, to verify protein contamination, and at 260/230 nm, to verify contamination by phenolic compounds or any other reagents used during the extraction process, has shown values >1.8, indicating the purity of the extracted RNA. Thereby, the RNA obtained with this method was intact, free of protein and organic compound contamination and free of genomic DNA, owing to the applied step of treatment with DNase. Thus, all the factors potentially affecting downstream analyses were virtually eliminated. As a matter of fact, we successfully analysed the expression of bone-specific genes (i.e. *SPP1*, *SOST* and *POSTN*) by RT-qPCR and relevant differences were highlighted between the two fractions.

Related to TRIzol-based protein extraction, protein pellet solubilization represents the main challenge, since, following this method, a packed protein pellet is produced. A non-complete solubilization could alter the correct protein estimation and may represent a limitation for downstream analysis. Indeed, several studies described alternative methods for protein solubilization in order to improve the yield [12,19,21].

In our study, protein solubilization was performed according to the TRIzol manufacturer's protocol, by suspending protein pellets in 1% SDS and by incubating pellets for 40 min at 50°C.

Protein of both high and low molecular weight was successfully extracted from both cortical and trabecular bone and proteins from the two compartments had consistently different distribution patterns. Immunoblotting analyses for some of the bone-specific proteins, such as OPN, SPARC, total OC and sclerostin, confirmed the stability and the maintenance of immune reactivity of the extracted proteins and confirmed the existence of a different expression profile in different bone compartments. Despite the reliability and the high quality and yield of total RNA and proteins obtained with this method, this study suffers from some limitations. The main one is the lack of comparison with other methods. Working with human samples suffers from limited availability of withdrawable specimens and their small size; therefore, the possibility of processing the same sample with different methods is, actually, limited. Moreover, in this study, long-term RNA and protein stability has not been investigated. This would represent another limit of the study as well as the lack of analysis of low expression/less stable proteins, high molecular weight proteins, membrane-bound proteins and those with a tendency to aggregate. Further studies would be needed to define whether storage conditions affect the stability of molecular species isolated from tissues that are difficult to handle, such as bone. However, one of our main goals was to demonstrate that bone homogenization, before isolation, does not represent a limiting step. In this regard, RNA and protein extraction from bone gave results that were absolutely comparable, in terms of quality and integrity, with those obtained from cultured cells (SaOS-2), whose processing does not require any homogenization. In conclusion, we describe a TRIzol-based multistep method for the simultaneous extraction of total RNA and protein, from the same human bone sample, divided *ex vivo* into the cortical and trabecular fractions. Overall, our results show that the yield and quality of extracted RNA and proteins are appropriate for downstream molecular studies and enable site-specific subtle differences to be revealed.

## Data Availability

The data that support the findings of this study are openly available as supplementary figures (western blot analyses) and on Zenodo (doi:10.5281/zenodo.5749901).

## References

[1] Hughes A, Stewart TL, Mann V. 2012 Extraction of nucleic acids from bone. Methods Mol. Biol. **816**, 249-259. (10.1007/978-1-61779-415-5_17) 22130934

[2] Cepollaro S, Della Bella E, de Biase D, Visani M, Fini M. 2018 Evaluation of RNA from human trabecular bone and identification of stable reference genes. J. Cell. Physiol. **233**, 4401-4407. (10.1002/jcp.26319) 29206301

[3] Parfitt AM. 2002 Misconceptions (2): turnover is always higher in cancellous than in cortical bone. Bone **30**, 807-809. (10.1016/S8756-3282(02)00735-4) 12052445

[4] Seeman E. 2013 Age- and menopause-related bone loss compromise cortical and trabecular microstructure. J. Gerontol. A Biol. Sci. Med. Sci. **68**, 1218-1225. (10.1093/gerona/glt071) 23833200

[5] Nickolas TL *et al**.* 2013 Rapid cortical bone loss in patients with chronic kidney disease. J. Bone Mineral. Res. **28**, 1811-1820. (10.1002/jbmr.1916) PMC3720694 23456850

[6] Ott SM. 2018 Cortical or trabecular bone: what's the difference? Am. J. Nephrol. **47**, 373-375. (10.1159/000489672) 29788030

[7] Mullegama SV, Alberti MO, Au C, Li Y, Toy T, Tomasian V, Xian RR. 2019 Nucleic acid extraction from human biological samples. Methods Mol. Biol. **1897**, 359-383. (10.1007/978-1-4939-8935-5_30) 30539458

[8] Carter LE, Kilroy G, Gimble JM, Floyd ZE. 2012 An improved method for isolation of RNA from bone. BMC Biotechnol. **12**, 5. (10.1186/1472-6750-12-5) 22260224 PMC3282642

[9] Tsangari H, Findlay DM, Kuliwaba JS, Atkins GJ, Fazzalari NL. 2004 Increased expression of IL-6 and RANK mRNA in human trabecular bone from fragility fracture of the femoral neck. Bone **35**, 334-342. (10.1016/j.bone.2004.02.006) 15207775

[10] Canciani E, Dellavia C, Marazzi MG, Augusti D, Carmagnola D, Vianello E, Canullo L, Galliera E. 2017 RNA isolation from alveolar bone and gene expression analysis of RANK, RANKL and OPG: a new tool to monitor bone remodeling and healing in different bone substitutes used for prosthetic rehabilitation. Arch. Oral Biol. **80**, 56-61. (10.1016/j.archoralbio.2017.03.011) 28384521

[11] Becker C, Hammerle-Fickinger A, Riedmaier I, Pfaffl MW. 2010 mRNA and microRNA quality control for RT-qPCR analysis. Methods **50**, 237-243. (10.1016/j.ymeth.2010.01.010) 20079844

[12] Wen Y, Vechetti Jr IJ, Valentino TR, McCarthy JJ. 2020 High-yield skeletal muscle protein recovery from TRIzol after RNA and DNA extraction. BioTechniques **69**, 264-269. (10.2144/btn-2020-0083) 32777951 PMC7566772

[13] Fleige S, Pfaffl MW. 2006 RNA integrity and the effect on the real-time qRT-PCR performance. Mol. Aspects Med. **27**, 126-139. (10.1016/j.mam.2005.12.003) 16469371

[14] Skrypina NA, Timofeeva AV, Khaspekov GL, Savochkina LP, Beabealashvilli R. 2003 Total RNA suitable for molecular biology analysis. J. Biotechnol. **105**, 1-9. (10.1016/s0168-1656(03)00140-8) 14511905

[15] Wilson TA, Kaur N, Davis J, Ali SA. 2021 Tissue collection and RNA extraction from the human osteoarthritic knee joint. J. Vis. Exp. **173**. (10.3791/62718) 34369924

[16] Kelly NH, Schimenti JC, Ross FP, van der Meulen MC. 2014 A method for isolating high quality RNA from mouse cortical and cancellous bone. Bone **68**, 1-5. (10.1016/j.bone.2014.07.022) 25073031 PMC4281890

[17] Pedersen KB, Williams A, Watt J, Ronis MJ. 2019 Improved method for isolating high-quality RNA from mouse bone with RNAlater at room temperature. Bone Rep. **11**, 100211. (10.1016/j.bonr.2019.100211) 31198821 PMC6558217

[18] Licini C, Montalbano G, Ciapetti G, Cerqueni G, Vitale-Brovarone C, Mattioli-Belmonte M. 2020 Analysis of multiple protein detection methods in human osteoporotic bone extracellular matrix: from literature to practice. Bone **137**, 115363. (10.1016/j.bone.2020.115363) 32298836

[19] Hummon AB, Lim SR, Difilippantonio MJ, Ried T. 2007 Isolation and solubilization of proteins after TRIzol extraction of RNA and DNA from patient material following prolonged storage. BioTechniques **42**, 467-472. (10.2144/000112401) 17489233 PMC4721573

[20] Likhite N, Warawdekar UM. 2011 A unique method for isolation and solubilization of proteins after extraction of RNA from tumor tissue using trizol. J. Biomol. Techniques. **22**, 37-44. PMC3059540 21455480

[21] Simoes AE et al. 2013 Efficient recovery of proteins from multiple source samples after TRIzol((R)) or TRIzol((R))LS RNA extraction and long-term storage. BMC Genomics **14**, 181. (10.1186/1471-2164-14-181) 23496794 PMC3620933

[22] Jiang X, Ye M, Jiang X, Liu G, Feng S, Cui L, Zou H. 2007 Method development of efficient protein extraction in bone tissue for proteome analysis. J. Proteome Res. **6**, 2287-2294. (10.1021/pr070056t) 17488005

[23] Cleland TP, Voegele K, Schweitzer MH. 2012 Empirical evaluation of bone extraction protocols. PLoS ONE **7**, e31443. (10.1371/journal.pone.0031443) 22348088 PMC3279360

[24] Cleland TP, Vashishth D. 2015 Bone protein extraction without demineralization using principles from hydroxyapatite chromatography. Anal. Biochem. **472**, 62-66. (10.1016/j.ab.2014.12.006) 25535955 PMC4314460

[25] Cleland TP. 2018 Solid digestion of demineralized bone as a method to access potentially insoluble proteins and post-translational modifications. J. Proteome Res. **17**, 536-542. (10.1021/acs.jproteome.7b00670) 29166020

[26] Piccoli A et al. 2020 Sclerostin regulation, microarchitecture, and advanced glycation end-products in the bone of elderly women with type 2 diabetes. J. Bone Mineral. Res. **35**, 2415-2422. (10.1002/jbmr.4153) PMC8143610 32777114

